# When should patients take simethicone orally before colonoscopy for avoiding bubbles: A single-blind, randomized controlled study

**DOI:** 10.1097/MD.0000000000033728

**Published:** 2023-05-12

**Authors:** Woohyuk Jung, Gyu Man Oh, Jae Hyun Kim, Youn Jung Choi, Min Young Son, Kyoungwon Jung, Sung Eun Kim, Won Moon, Moo In Park, Seun Ja Park

**Affiliations:** a Department of Internal Medicine, Kosin University College of Medicine, Busan, Korea.

**Keywords:** antifoaming agents, bowel preparation solution, colonoscopy, polyethylene glycol, simethicone

## Abstract

Many studies have reported the use of simethicone before colonoscopy removes bubbles. However, guidelines weakly recommend simethicone administration before colonoscopy. The present study aimed to confirm the advantages of taking simethicone and determine the appropriate time for taking simethicone.

**Methods:**

We randomly assigned patients to the following 5 groups according to the administration time: 4 groups were divided based on 2 parameters (the day before and on the day of colonoscopy and before and after bowel cleansing) and the remaining group was the control group. We compared bubble score (BS), number of simethicone solution irrigations when visually obscured, satisfaction score of the endoscopist, insertion time.

**Results:**

A total of 204 patients were included in the study. There was a difference in BS according to the timing of simethicone administration (*P* < .001). The group taking simethicone on the day of the test had a better BS than the group taking simethicone the day before (*P* < .001). The group taking simethicone on the previous day had a better BS than the control group (*P* = .001). In the group of taking simethicone on the examination day, the number of irrigations was lower, and satisfaction with the inspector was higher than group of taking simethicone on previous day and control group (both *P* < .001). The insertion time showed a non-significantly decreasing trend (*P* = .417).

**Conclusion:**

Administering simethicone reduced bubbles and facilitated effective colonoscopy, especially when administrating it on the day of examination. It needs to be administered on the day of the examination regardless of bowel preparation.

## 1. Introduction

Colonoscopy is a useful tool for detecting and removing colorectal polyps, which is a precursor lesion of colorectal cancer.^[1]^ High-quality bowel preparation is essential for a thorough observation of the colon.^[2]^ For bowel preparation, laxatives such as polyethylene glycol (PEG), magnesium citrate plus picosulfate, and oral sulfate are consumed with water.^[3]^ During the bowel cleansing process, many bubbles are generated because a large amount of water mixed with mucus and bile.^[4]^ Endoscopists encounter a new obstacle -bubbles- even in the absence of stool.

Many studies have shown that oral administration of simethicone before colonoscopy helps eliminate these bubbles.^[5–8]^ Simethicone removes bubbles by reducing the surface tension of water. Two studies on the optimal time for simethicone administration during bowel cleansing with split-dose PEG reported that taking simethicone the day before the test is the best approach to reduce bubbles in the colon.^[9,10]^ However, these previous studies had limitations in that the administration time of simethicone was approximately divided as on the day before and on the day of colonoscopy. It is still questionable how to apply the results of these studies to actual clinical situations because patients still do not know when to take it exactly.

In addition, simethicone administration can reduce bloating symptoms.^[11]^ Bloating is a common side effect of patients who drink large amounts of liquid for colonoscopy preparation. Also, the price is low. Despite these merits, guidelines still weakly recommend taking simethicone orally before colonoscopy, and many clinicians have not been convinced.^[3]^ Many digestive endoscopy centers have not yet administered simethicone orally to all patients before colonoscopy.

Therefore, this study aimed to confirm the advantages of taking simethicone compared to those by not taking it and attempted to determine the optimal time for taking simethicone during bowel preparation to reduce bubbles.

## 2. Patients/material and methods

### 2.1. Study overview

This was a prospective, single-blind, randomized controlled study conducted in a single medical center. This study was approved by the institutional review board of Kosin University Gospel Hospital (approval no.: 2021-03-040). This trial was registered on the International Clinical Trials Registry Platform (number KCT0006782).

### 2.2. Patients

Patients older than 19 years who were hospitalized for colonoscopy examination for screening or post-polypectomy surveillance at the digestive endoscopy center of Kosin University Gospel Hospital were included. Exclusion criteria are as follows: under 19 years of age, pregnant women, requiring emergency colonoscopy due to hematochezia, melena and obstruction, changes in alimentary tract due to previous surgery, inflammatory bowel disease, suspected intestinal stenosis, bowel preparation, conditions predicted to fail bowel preparation. Patients with issues regarding the side effects of PEG and simethicone (chronic kidney disease, heart failure, history of drug allergy, etc) were also excluded.

### 2.3. Study design

After the patients were hospitalized, they underwent bowel cleansing by drinking 2 L of PEG plus ascorbic acid solution using the split-dose method. Briefly, 1 L of PEG plus ascorbic acid solution was administered in 2 doses, that is, once the night before the test and on the morning of the day of the test. Patients were divided into 5 groups according to the simethicone administration time: 4 experimental groups and 1 control group. Patients were randomized to each of the experimental group. The control group did not receive simethicone treatment. The experimental groups were divided into the following subgroups according to the timing of simethicone administration: the day before the examination and before bowel cleansing (BB); the day before the examination and after bowel cleansing (BA); the day of the examination and before bowel cleansing (TB); and the day of the examination and after bowel cleansing (TA). Simethicone was administered orally at a dose of 200 mg (10 mL). For bowel cleansing, patients drank 1 L of solution for 2 hours from 8 PM to 10 PM the day before the test and drank the remaining 1 L for 2 hours from 6 AM to 8 AM on the day of the test. Because the patients only needed to drink the solution in a set amount of time, the start and end times could not be controlled equally for everyone (see Fig., http://links.lww.com/MD/I947, Supplemental Content, which illustrates the experimental design).

Colonoscopy was performed between 11 AM and 4 PM. Before the test, the patients completed a questionnaire about the time to take simethicone, the time to finish taking a bowel cleansing agent, and adverse effects after taking simethicone. The endoscopist who performed the colonoscopy was blinded to the time of simethicone administration of the patient. Five experts who performed more than 2000 cases in total and more than 300 cases per year performed colonoscopy examinations. The bubble score (BS) scale was used to evaluate the number of bubbles, which is as follows: 3 (<5% of bubbles covering mucosa, no obscuration), 2 (5%–25% of bubbles covering the mucosa, mild obscuration), 1 (25%–50% of bubbles covering the mucosa, moderate obscuration), 0 (>50% of bubbles covering the mucosa, severe obscuration).^[4]^ The Aronchick Bowel Preparation Score (ABPS) was used to assess bowel preparation quality as follows: excellent; good; fair; poor; and inadequate.^[12]^ If there was no visibility due to bubbles, then the endoscopists irrigated 50 mL of a simethicone-mixed solution (20 mL of simethicone plus 30 mL of normal saline) through a biopsy channel to remove the bubbles.

After the colonoscopy examination, the endoscopists recorded BS, ABPS, and the number of irrigations of the simethicone-mixed solution for the right colon, transverse colon, and left colon. They also recorded the satisfaction score of patients’ bowel preparation with a numeric rating (0–10, 0: very bad, 10: excellent). To reduce variation and equalize the evaluation criteria among endoscopists, a guideline paper was attached to every endoscopy room, and the endoscopist performed calibration exercises on scoring systems before and after the examination (see Fig., http://links.lww.com/MD/I947, Supplemental Content).

### 2.4. Outcomes

The primary outcomes were BS and the secondary outcomes were total number of irrigations, endoscopist satisfaction score, ABPS, polyp detection rate, adenoma detection rate, insertion time, and withdrawal time.

### 2.5. Statistical analysis

In previous studies, simethicone administration improved the BS by approximately 30% to 50%.^[6,9]^ There was a difference of 2% to 40% in BS between the groups on the day before the colonoscopy and on the day of the colonoscopy.^[9,10]^ Based on these study results, the difference in BS between groups was estimated to be 25%. The number of patients required was at least 40 for each group with a power of 0.8, and an alpha value of 0.05. Considering a dropout rate of 10%, 220 patients (44 patients in each group) were required for the study. To compare the 2 groups, Student *t* test and the chi-square test were conducted for continuous and categorical variables as appropriate, and to compare 5 groups, a 1-way ANOVA analysis was conducted. Logistic regression analysis was performed to identify other factors affecting the BS. Statistical significance was set at *P* < .05. Statistical analyses were performed using SPSS, version 28.0 (IBM Co., Armonk, NY).

## 3. Results

### 3.1. Baseline characteristics

**Figure 1.**
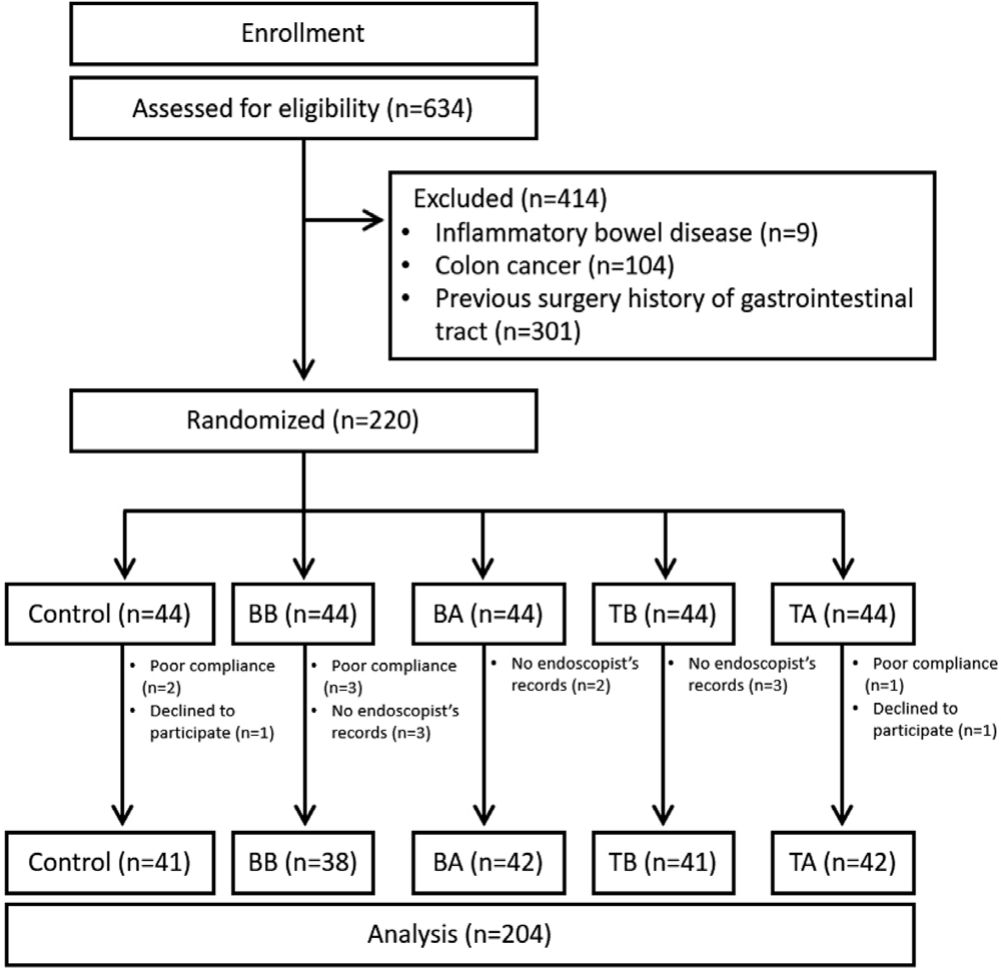
Study flow chart.

A total of 220 patients were enrolled between June and November 2021 (Fig. 1). Sixteen patients dropped out of the study (poor compliance, n = 6; declined to participate, n = 2; no endoscopist records, n = 8) and 204 patients were finally included. The mean age was 60.33 years old, and 105 patients were male (Table 1). “Presence of symptoms” were the most common reason to perform colonoscopy (n = 93, 45.59%). Among the symptoms, abdominal pain was the most common in 36 (38.71%) patients, followed by dyspepsia in 16 (17.20%) and anemia in 14 (15.05%). Most of them reported that it was easy to take simethicone (n = 160, 78.82%), and some patients complained of nausea (n = 32, 15.69%).

**Table 1:** Patient clinical characteristics and difficulty in taking simethicone.

Characteristics	Patient data (N = 204)
Age, yr (range)	60.33 ± 12.65 (19–87)
Male:female	105:99
Height, cm	163.2 ± 8.94
Weight, kg	65.07 ± 11.47
Indication for colonoscopy	
Screening	51
Post-polypectomy surveillance	60
Presence of symptoms	93
Symptoms	
Abdominal pain	36
Dyspepsia	16
Anemia	14
Diarrhea	10
Constipation	6
Fecal incontinence	6
Weight loss	4
Insertion time, median (IQR*)	4.87 (3.02–7.65)
Withdrawal time, median (IQR)	17.97 (11.50–28.87)
Bubble score	5.98 ± 2.61
Aronchick bowel preparation scale	7.12 ± 2.61
Polyp detection rate (%)	77.61
Adenoma detection rate (%)	68.16
Difficulty in taking simethicone	
Ease	126
Relative ease	34
Slightly difficult	41
Quite difficult	0
Tolerability	
Abdominal pain	0
Vomiting	0
Nausea	32
Abdominal distension	9

* IQR, interquartile range.

There was a clear difference in the time of simethicone administration between the groups (see Table, http://links.lww.com/MD/I948, Supplemental Content, which shows the time of taking simethicone). However, the standard deviation of the administration time on the day before the test was relatively larger than that on the day of the test.

### 3.2. Primary outcome

**Figure 2.**
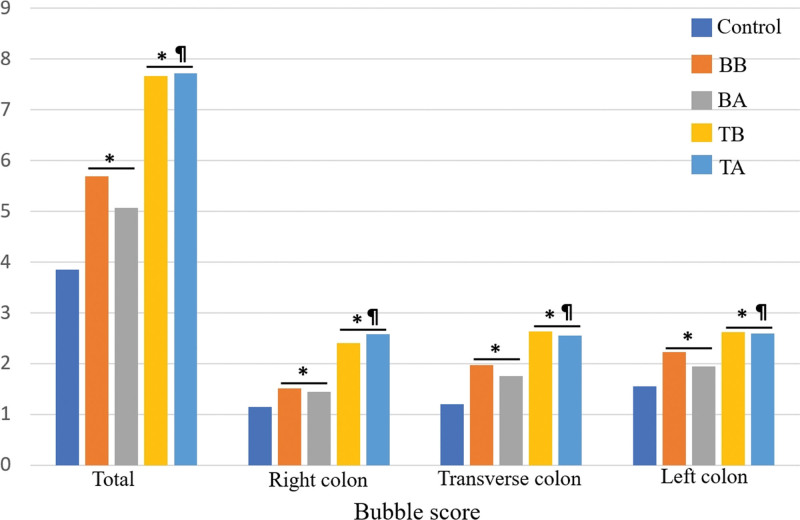
Comparison of bubble scores: In all parts of the colon, BB and BA had better bubble scores than the control group. Also, TB and TA were better than BB and BA. However, there was no difference between TB and TA group. *P < .05, compared to control group. ¶*P* < .05, compared to BB and BA group. BA = the day before the examination and after bowel cleansing, BB = the day before the examination and before bowel cleansing, TA = the day of the examination and after bowel cleansing, TB = the day of the examination and before bowel cleansing.

There was a significant difference in the BS according to the timing of simethicone administration (*P* < .001) (Table 2). The group taking simethicone on the day of the test had a better BS than the group taking simethicone the previous day (Fig. 2). The group taking simethicone on the previous day had a better BS than the control group. However, there was no difference in BS between the TB and TA groups, nor between TB and TA. When the colon was divided into 3 parts (right, transverse, left), BS tended to increase. In each 3 parts of colon, like total BS results, TB and TA groups had better BS than BB, BA, and control group. But no differences between TB and TA groups.

**Table 2:** Clinical outcomes.

	Control	BB†	BA‡	TB§	TA¶	*P* value
Bubble score, mean						
Right colon	1.150	1.513	1.450	2.410	2.575	<.001
Transverse colon	1.200	1.974	1.750	2.641	2.550	<.001
Left colon	1.550	2.231	1.950	2.615	2.600	<.001
Total	3.850	5.692	5.075	7.667	7.725	<.001
Number of irrigations, mean						
Cecum	0.875	0.675	0.550	0.200	0.150	<.001
Ascending colon	0.950	0.650	0.475	0.025	0.150	<.001
Transverse colon	0.600	0.300	0.450	0.050	0.100	<.001
Descending colon	0.150	0.025	0.225	0.000	0.000	.001
Sigmoid colon and rectum	0.050	0.075	0.075	0.000	0.000	.318
Total	2.600	1.725	1.775	0.275	0.400	<.001
Satisfaction score, mean	5.175	6.300	6.077	8.400	7.925	<.001
Insertion time, mean, min	7.167	6.483	6.083	5.700	5.317	.417
Withdrawal time, mean, min	19.95	19.95	25.05	18.80	23.30	.179
Aronchick bowel preparation score, mean						
Right colon	2.675	2.400	2.725	2.180	2.450	.049
Transverse colon	2.600	2.450	2.600	1.923	2.150	.004
Left colon	2.525	2.325	2.500	1.923	2.150	.016
Total	7.800	7.175	7.825	6.026	6.750	.009
Polyp detection rate (%)						
Cecum	25.0	27.5	15.0	15.0	30.0	.337
Ascending colon	40.0	55.0	47.5	35.0	37.5	.364
Transverse colon	37.5	47.5	47.5	32.5	37.5	.566
Descending colon	12.5	17.5	22.5	20.0	25.0	.676
Sigmoid colon and rectum	37.5	42.5	52.5	50.0	45.0	.687
Total	75.0	85.0	77.5	75.0	77.5	.818
Adenoma detection rate (%)						
Cecum	22.5	17.5	7.7	5.4	29.0	.013
Ascending colon	32.5	42.5	41.0	29.7	34.2	.705
Transverse colon	27.5	35.0	30.8	24.3	21.1	.721
Descending colon	7.5	17.5	18.0	10.8	15.8	.473
Sigmoid colon and rectum	35.0	35.0	35.9	40.5	34.2	.819
Total	65.0	75.0	69.2	62.2	65.8	.796

† BB = the day before the examination and before bowel cleansing.

‡ BA = the day before the examination and after bowel cleansing.

§ TB = the day of the examination and before bowel cleansing.

¶ TA = the day of the examination and after bowel cleansing.

### 3.3. Secondary outcome

**Figure 3.**
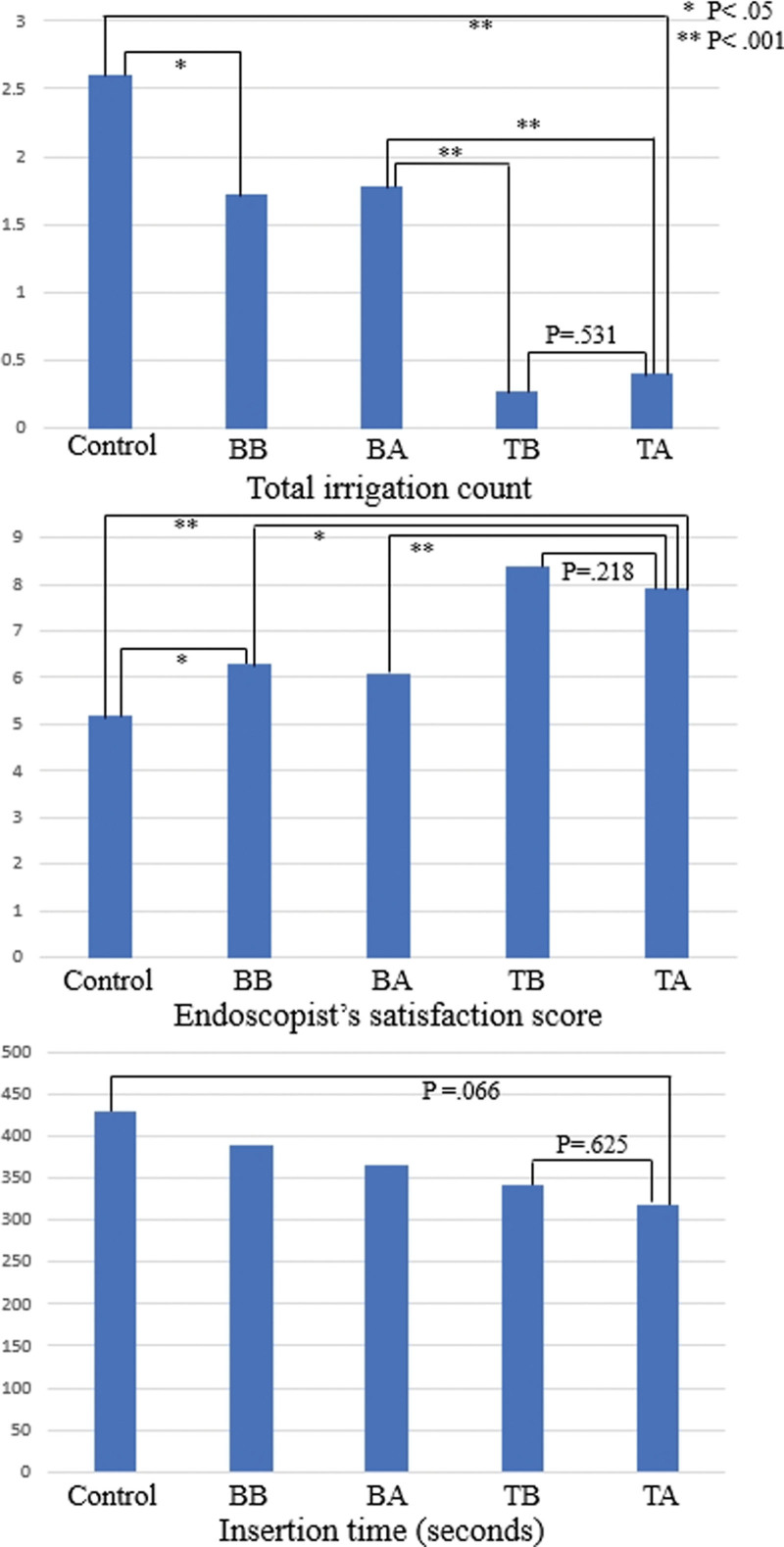
Comparison of total irrigation count, endoscopist satisfaction score, and insertion time: Total irrigation count and endoscopist satisfaction score of BB and BA group were better than that of control group. Also, TB and TA group had better results than BB and BA group. There was no difference between TB and TA group. Insertion time tended to decrease overall, but there was no significant difference. BA = the day before the examination and after bowel cleansing, BB = the day before the examination and before bowel cleansing, TA = the day of the examination and after bowel cleansing, TB = the day of the examination and before bowel cleansing.

The number of irrigations was significantly lower and the satisfaction of the inspector was higher, similar to the result seen for BS (Fig. 3). Similar results were observed even when the colon was subdivided into 3 parts. As it came to the distal part, the number of irrigations tended to decrease. The ABPS was better in the TB and TA groups than in the BB, BA, and control groups. However, there was no difference between the groups regarding simethicone administration on the day before colonoscopy and the control group (Table 2). Even when the colon was divided into 3 parts as right colon, transverse colon, and left colon, and ABPS was observed, there was no clear trend, but the *P* value was significantly different (right colon, 0.049; transverse colon, 0.004; left colon, 0.016). Polyp detection rate and adenoma detection rate showed no significant difference. However, the insertion time tended to decrease with the administration of simethicone close to the colonoscopy examination, but the difference was not significant (*P* = .417). There was no difference in withdrawal time.

### 3.4. Factors associated with BS

Regarding factors that can affect BS, the time interval from bowel preparation to colonoscopy, the time interval from taking simethicone to colonoscopy, and the level of bowel preparation were considered. A good BS was defined as a total BS score of 7 or higher, and the appropriate time interval from taking simethicone to colonoscopy was defined as <11 hours (i.e., taking simethicone on the day of colonoscopy). Good bowel preparation was defined as a total ABPS score of 8 or less, and the appropriate time interval from bowel preparation to colonoscopy was taken between 3 and 7 hours based on the previous study result.^[13]^ The time interval from taking simethicone to colonoscopy affects the BS the most (odds ratio: 5.178, 95% confidence interval: 2.331–11.504) (Table 3). However, good bowel preparation also influences BS. Although negative results were obtained, the odds ratio of the time interval from bowel preparation to colonoscopy was 1.990 (95% confidence interval: 0.894–4.430, *P* = .092).

**Table 3:** Regression analysis of factors associated bubble score.

	Odds ratio (95% CI)	*P* value
Simethicone administration time (taking simethicone on the day of colonoscopy)	5.317 (2.401–11.778)	<.001
Total ABPS† <9	4.542 (1.1992–10.360)	<.001
Bowel preparation to colonoscopy interval (3–7 hr)	1.990 (0.894–4.430)	.092

† ABPS = Aronchick Bowel Preparation Score, CI = confidence interval.

## 4. Discussion

This prospective, single-center, single-blind, randomized controlled study aimed to determine the optimal timing for simethicone administration along with 2 L of PEG plus ascorbic acid solution to reduce the number of bubbles formed during colonoscopy. Administering simethicone was found to reduce bubbles significantly when compared to not taking it, and taking it on the day of colonoscopy reduced more bubbles than taking it the day before the examination. The number of bubbles did not change taking simethicone before or after bowel cleansing as long as it was taken on the day of colonoscopy.

There have been many previous studies on the effects of oral administration of simethicone before colonoscopy. A study showed that taking simethicone significantly reduced bubbles,^[4]^ while a study was conducted to determine the most effective dose of simethicone^[14]^ and another study was conducted to determine the most effective timing of taking simethicone.^[9,10]^ Also, there was a study that posed mixing simethicone with 2 L of PEG-ascorbic acid solution for both bowel cleansing and eliminating bubbles was better.^[5]^ There is no clearly established protocol to date, but when the results of previous studies are combined, it can be thought that taking 200 mg of simethicone on the day before the examination is most effective. Whereas previous studies suggested that taking it the day before was better, the results of this study found the exact opposite: taking it on the day of the test had better BS.

The digestive endoscopy center of the authors empirically prescribed 200 mg of simethicone on the day of examination and after the completion of bowel cleansing, and we had a decent BS in the usual colonoscopy tests unlike previous studies’ results. Therefore, we attempted to determine the best timing and found that taking simethicone on the day of colonoscopy is more effective than taking it the day before the examination. When the simethicone was taken the day before, 1 L of bowel cleansing solution was added on the day of the test, and this led to the formation of bubbles again.

Before the study, we hypothesized that taking simethicone before preparation on the day of the test would be the most effective approach. The reason is that colonoscopy is performed 3 to 5 hours after taking the last bowel cleansing solution, and we thought that a small amount of simethicone (10 mL) would not pass through the alimentary tract during that time. So, we anticipated the “TB” group would have better BS than “TA” group. However, on the day of endoscopy, there was no significant difference depending on the administration time in this present study. This is probably because the number of bubbles was influenced not only by the timing of simethicone administration but also by the level of bowel cleansing itself. In other words, patients with poor bowel preparation scores also had poor BS. Although it had null data, the effect of the time between the completion of bowel preparation and the colonoscopy did not seem to be negligible.

Although simethicone is not expensive, many endoscopy centers still do not pay much attention to taking simethicone as much as bowel cleansing medicine (price, patient education, checking patient compliance, etc). However, taking simethicone before colonoscopy clearly reduced BS and increased endoscopist satisfaction in this study. In addition, simethicone irrigation was forced to remove remaining bubbles, but when taken simethicone in advance, the total amount of use including irrigation was also reduced. In this study, if endoscopists used 20 mL of simethicone per once for irrigation to reduce bubbles, then approximately 52 mL of simethicone was used in the control group, 35 mL in the BB and BA groups, and 6.75 mL in the TB and TA groups. It is also recommended to take simethicone on the day of the test in a cost-effective manner. As expected, and as reported in previous studies and in this study, there were no serious side effects of simethicone.

The strength of this study is that, based on the results of a previous study, the administration time was divided in more detail and clearly. Second, because all participants prepared for colonoscopy during hospitalization, compliance with bowel cleansing and simethicone medication was guaranteed. Third, unlike other studies, the frequency of simethicone irrigation was analyzed to determine the difference in the amount of simethicone used between patients treated with simethicone and those who did not.

However, this study has several limitations. First, the subjects were randomized into 5 groups with administration timing, but randomization of 5 colonoscopy operators was not performed. Therefore, the same evaluation criteria may not have been applied in each group. Each endoscopist may have different scoring criteria; therefore, we tried to reduce this difference by attaching a guideline paper to each endoscopy room. However, the endoscopist tendency to decide on simethicone irrigation based on the number of bubbles could vary. Second, in this study, BS and ABPS improved together, but the causal relationship could not be clearly defined. Endoscopists were asked to consider BS and ABPS separately, but both had the same tendencies. It seemed that the BS and the ABPS themselves would have an effect on each other and would also affect the satisfaction score. Third, because of the small number of samples, it was not possible to analyze which time period would be the best on the day of colonoscopy. A follow-up study is required in this regard.

## 5. Conclusion

In conclusion, administering simethicone before colonoscopy significantly reduced bubbles and facilitated colonoscopy. In particular, when taking simethicone on the day of the colonoscopy regardless of bowel cleansing completion, the bubble removal effect was greater than taking it the day before the colonoscopy. This is helpful for effective colonoscopy, and the procedure is cost-effective.

## Author contributions

**Conceptualization:** Gyu Man Oh, Won Moon.

**Data curation:** Jae Hyun Kim, Youn Jung Choi, Min Young Son.

**Investigation:** Jae Hyun Kim, Sung Eun Kim.

**Methodology:** Jae Hyun Kim.

**Supervision:** Seun Ja Park.

**Validation:** Moo In Park.

**Visualization:** Gyu Man Oh, Kyoungwon Jung.

**Writing – original draft:** Woohyuk Jung, Gyu Man Oh.

**Writing – review & editing:** Woohyuk Jung, Gyu Man Oh, Kyoungwon Jung, Sung Eun Kim, Won Moon, Moo In Park, Seun Ja Park.

## Supplementary Material




