# Supplementary material for: When should patients take simethicone orally before colonoscopy for avoiding bubbles: A single-blind, randomized controlled study
Source: Medicine (Baltimore). 2023 May 12;102(19):e33728. doi: 10.1097/MD.0000000000033728 (PMC10174409; doi:10.1097/MD.0000000000033728)

Supplement figure. Study design and bubble score scale

(A): 3 (<5% of bubbles covering mucosa, no obscuration), (B): 2 (5-25% of bubbles covering the mucosa, mild obscuration), (C): 1 (25-50% of bubbles covering the mucosa, moderate obscuration), (D): 0 (>50% of bubbles covering the mucosa, severe obscuration)

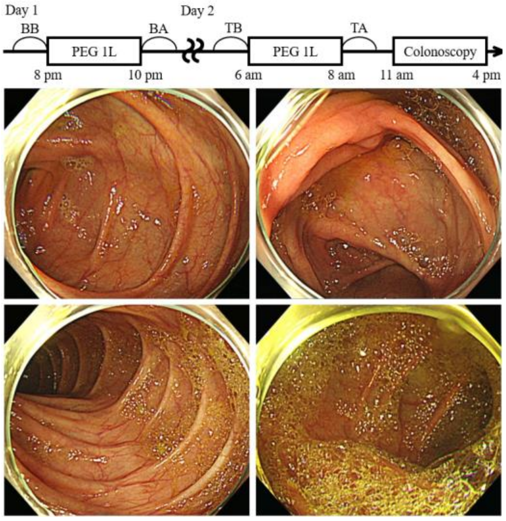

Supplement: Supplementary file 1 [file medi-102-e33728-s001.pdf]
