# Supplementary material for: When should patients take simethicone orally before colonoscopy for avoiding bubbles: A single-blind, randomized controlled study
Source: Medicine (Baltimore). 2023 May 12;102(19):e33728. doi: 10.1097/MD.0000000000033728 (PMC10174409; doi:10.1097/MD.0000000000033728)
Supplement: Supplementary file 2 [file medi-102-e33728-s002.pdf]

Supplement

Supplement table. Average administration time of simethicone in each group.

Group	Average administration time	Standard deviation	p-value
BB	20:54 the day before the test	3 h 56 min	<0.001
BA	22:38 the day before the test	3 h 43 min	
TB	05:39 on the day of the test	2 h 56 min	
TA	08:27 on the day of the test	1 h 59 min	

BB, the day before the examination and before bowel cleansing; BA, the day before the examination and after bowel cleansing; TB, the day of the examination and before bowel cleansing; TA, the day of the examination and after bowel cleansing
